# ω3 Polyunsaturated Fatty Acids as Immunomodulators in Colorectal Cancer: New Potential Role in Adjuvant Therapies

**DOI:** 10.3389/fimmu.2016.00486

**Published:** 2016-11-15

**Authors:** Stefania Miccadei, Roberta Masella, Anna Maria Mileo, Sandra Gessani

**Affiliations:** ^1^Unit of Tumor Immunology and Immunotherapy, Department of Research, Advanced Diagnostic and Technological Innovation, Regina Elena National Cancer Institute, Rome, Italy; ^2^Department of Veterinary Public Health and Food Safety, Istituto Superiore di Sanità, Rome, Italy; ^3^Department of Hematology, Oncology and Molecular Medicine, Istituto Superiore di Sanità, Rome, Italy

**Keywords:** ω3 polyunsaturated fatty acids, immunomodulation, colorectal cancer, adjuvant therapy, inflammation

## Abstract

Diet composition may affect the onset and progression of chronic degenerative diseases, including cancer, whose pathogenesis relies on inflammatory processes. Growing evidence indicates that diet and its components critically contribute to human health, affecting the immune system, secretion of adipokines, and metabolic pathways. Colorectal cancer (CRC) is one of the leading causes of death worldwide. Antineoplastic drugs are widely used for CRC treatment, but drug resistance and/or off-target toxicity limit their efficacy. Dietary ω3 polyunsaturated fatty acids (PUFA) have been gaining great interest in recent years as possible anti-inflammatory and anticancer agents, especially in areas such as the large bowel, where the pro-inflammatory context promotes virtually all steps of colon carcinogenesis. Growing epidemiological, experimental, and clinical evidence suggests that ω3 PUFA may play a role in several stages of CRC management exhibiting antineoplastic activity against human CRC cells, improving the efficacy of radiation and chemotherapy, ameliorating cancer-associated secondary complications, and preventing CRC recurrence. These effects are most likely related to the immunomodulatory activities of ω3 PUFA that are able to influence several aspects of the inflammatory process ranging from inflammasome activation, leukocyte recruitment, production of immune mediators to differentiation, and activation of immune cells. In this review, we will focus on the potential use of ω3 PUFA as adjuvant agents together with chemo/radiotherapy, highlighting the immunomodulatory effects most likely responsible for their beneficial effects in different stages of CRC management.

## Introduction

It has become increasingly clear that dietary habits may affect the risk/progression of chronic diseases with a pathogenic inflammatory component, such as colorectal cancer (CRC) (1). CRC is the third most common cancer and the fourth leading cause of cancer death worldwide and represents a clinical model of cancer showing a close relationship with inflammation and environmental factors (2). Inflammation is nowadays considered an hallmark of cancer (3), and exacerbated inflammatory processes has been reported to favor the insurgence of several types of neoplastic diseases, their progression, as well as a worse prognosis for the patient with cancer (World Cancer Report, 2014, IARC). Several types of inflammation differing by cause, mechanism, outcome, and intensity can promote cancer development and progression. Interestingly, rising CRC incidence trends are mainly regarded as a part of the change in lifestyle, including calorically excessive high-fat/low-fiber diet, lack of physical activity, and obesity (4). Epidemiological and clinical investigations have consistently evidenced a significant relationship between obesity-driven inflammation and most steps of CRC development, including initiation, promotion, progression, and metastasis (1). Furthermore, cancer therapy as well as surgery for cancer resection can trigger an inflammatory response by causing trauma, necrosis, and tissue injury that stimulate tumor re-emergence and resistance to therapy (5). In spite of the progress made in recent years in cancer treatment, the clinical relevance of the conventional therapeutic modalities alone in solid tumors, including CRC, still pose major questions due to the high costs of drugs and unsatisfactory long-term clinical results (6). Thus, due to the impact of inflammation as a risk factor for CRC development as well as in the course of the disease, new concepts are emerging on the benefits that may derive from the combination of conventional CRC treatments with intervention strategies targeted at inhibiting/attenuating chronic inflammation. Notably, dietary components influence inflammatory processes including intestinal inflammation, and dietary habits have been suggested to play a major role in CRC risk (7). Considerable attention has therefore been directed toward the ability of nutritional agents to target key molecular pathways involved in cancer-related inflammation. It has only recently gained acceptance that fatty acids (FAs) are major determinants in inflammation and may influence the risk of related pathologies. Given their precursor status to signaling lipid mediators, FAs act as ligands for key immune receptors, and their activation can initiate and perpetuate an innate immune response (8).

## Dietary Polyunsaturated Fatty Acids

ω3 and ω6 FAs are polyunsaturated fatty acids (PUFA) whose definition derives from counting the carbon atoms after the last double bond in the end of the fatty acid chain (9). The type of response they induce strongly depends on their biochemical properties, mainly related to number and position of double bonds (10). In this regard, ω3 and ω6 PUFA exhibit different behaviors with respect to the inflammatory process, exerting anti-inflammatory or pro-inflammatory activity, respectively. Owing to the proposed competitive role of ω3 and ω6 PUFA, their dietary composition has been suggested to be a biologically plausible target for CRC management. ω3 and ω6 FAs represent naturally occurring substances required for biological process with key roles in phospholipid membrane structure and function, cellular signaling, and lipid metabolism (11, 12). In humans, the ω3 α-linolenic acid (ALA, 18:3ω3) as well as the ω6 linoleic acid (LA, 18:2ω6), precursors of several PUFA, are essential FA that cannot be synthesized by humans and must therefore be assumed from dietary sources including fish, flaxseeds, walnuts, and algae (9). Several kind of food, rich in ω3 PUFA, are typical of the so-called Mediterranean diet (MD). Several epidemiological studies have evaluated the effects of this dietary pattern as protective against several diseases associated with chronic low-grade inflammation such as cancer, diabetes, obesity, atherosclerosis, metabolic syndrome, and cognition disorders (13). Adherence to traditional MD was found to be associated with markedly and significantly reduced incidence of cardiovascular diseases and overall cancer (14, 15). Due to the poor endogenous conversion of ALA to its longer and more highly desaturated FAs, there is considerable debate as to whether the longer versions such as eicosapentaenoic acid (EPA, 20:5ω3) and docosahexaenoic acid (DHA, 22:6ω3) may be considered semi-essential (16). These PUFA are key components of cell membrane, lipoproteins, and adipose tissue (AT) whose composition closely reflects the dietary intake (17). For several years, ω3 PUFA have been studied extensively for the prevention of cardiovascular diseases (18, 19). More recently, their capacity to exert anticancer activity has been recognized. A plethora of studies pointed to ω3 PUFA and their oxidative metabolites as potential anti-inflammatory and anticancer agents, especially in areas such as the large bowel, where the influence of orally introduced substances is high and tumors show deranged PUFA patterns. In particular, modified ω3 and ω6 PUFA profiles have been found in serum and cancer tissue of CRC patients, suggesting possible alteration of PUFA metabolism that may play a role in inflammation-driven colorectal carcinogenesis (20, 21). Overall, these studies suggested that the metabolism of PUFA plays a role in inflammation-driven colorectal carcinogenesis and is likely influenced by the tumor size. In this regard, it is of relevance that ω3/ω6 PUFA ratio of the industrialized diets is imbalanced, and this condition results in a well-known pro-inflammatory effect (22). Noteworthy, a decreased ω3/ω6 PUFA ratio of visceral AT correlates with AT inflammatory status in CRC patients suggesting that qualitative, more than quantitative, PUFA changes in AT may influence tissue dysfunctions potentially linked to inflammatory conditions (23). Likewise, the sum of some PUFA [i.e., gamma linolenic acid (GLA), Di-homo-gamma linolenic acid (DGLA), and arachidonic acid (AA)] in both visceral and subcutaneous AT was found statistically higher in cancer patients with respect to healthy controls (24). Overall, these observations support the concept that PUFA derangement occurs not only in tumor tissue but also at microenvironment and systemic levels most likely contributing to tumor-associated inflammation.

There is now sufficient evidence from prospective studies indicating that fish consumption or fish oil ω3 PUFA intake is inversely related to CRC incidence in the general population (25). However, the results achieved in a recent meta-analysis carried out to evaluate the effects of ω3 PUFA on inflammatory markers in CRC patients highlighted that the beneficial effects of ω3 PUFA rely on supplementation protocols (e.g., dose, duration, and route of administration), the concomitant anticancer regimen adopted, as well as on the confounding effects due to other nutrients that might be present in the supplement (26). In this regard, it should be taken into account that the observational studies carried out to evaluate the potential anticancer effects of ω3 PUFA might have been limited by the insufficient homogeneity of the observations. An explanation for such heterogeneity relies likely on the inherent difficulties associated with epidemiology, including the confounding and dietary pattern context, measurement error, level of intake, and genetic polymorphisms (26, 27).

Despite the majority of studies has proven that ω3 PUFA administration is not toxic or carcinogenic, it is hard to imagine that these compounds could be exploited alone in the treatment of cancer. However, growing epidemiological, experimental, and clinical evidence showing the beneficial effects of ω3 PUFA in several stages of CRC management represent a solid rationale for their exploitation in therapeutic combinatory strategies (28). The antineoplastic effects of ω3 PUFA have been studied for many years, and multiple biological mechanisms have been suggested to explain their inhibitory effects on cancer cell growth (29, 30). However, in recent years, growing attention has been given to the antineoplastic activity of ω3 PUFA related to the action of these bioactive molecules on inflammatory process, due to the pathogenic role of inflammation in the development of many kinds of tumors. ω3 PUFA, particularly EPA and DHA, have emerged as nutrients capable of modulating both metabolic and immune processes. As summarized in Table 1, these PUFA are able to modulate a number of aspects of the inflammatory process ranging from inflammasome activation, leukocyte recruitment, and production of immune mediators, to differentiation and activation of immune cells. ω3 PUFA effects are mediated by different mechanisms including both extracellular (e.g., GPR120) (31) and intracellular (e.g., PPARγ) receptors that control inflammatory cell signaling and gene expression (32). In spite of many efforts, it remains to be fully defined how these compounds succeed in influencing such a variety of molecular pathways and cell/tissue functions with pleiotropic beneficial effects, and how these mechanisms are interconnected. Some of the ω3 PUFA-mediated events appear to rely, at least in part, on changes in FA composition of membrane phospholipids, relevant for the production of lipid mediators, and formation of lipid rafts in response to inflammatory stimuli (33).

**Table 1:** **Immunomodulatory effects of ω3 PUFA in monocyte/macrophages and T lymphocytes**.

ω3 PUFA exposure	Experimental/animal model	Observed effect	Suggested mechanism	Reference
DHA^a^, EPA^a^	MU and HU monocytic cell lines; MU and HU primary monocyte/Mφ, C57BL/6 mice	↓ inflammasome activation	GPR120-β arrestin-2-mediated NF-kB inhibition	(34–36)
			Enhanced autophagy	
			Impaired TLR2/TLR1 dimerization	
Isocaloric HFD-containing 27% menhaden fish oil (16% EPA, 9% DHA); isocaloric ω3 PUFA-enriched diet (3% menhaden fish-oil + 7% safflower oil)	C57BL/6 mice	↓ leukocyte chemotaxis	GPR120, β arrestin-2	(37, 38)
			NF-kB and STAT3 inhibition	
DHA^a^, EPA^a^	MU monocytic cell lines and primary Mφ	↑ efferocytic activity of Mφ	PPARγ and AKT activation	(39, 40)
DHA^a^, EPA^a^, isocaloric HFD-containing 27% menhaden fish oil (16% EPA, 9% DHA); HFD + DHA^a^	MU monocytic cell lines and primary Mφ	↑ M2 Mφ polarization	GPR120, β arrestin-2	(37, 40–42)
	C57BL/6 mice			
DHA^a^, EPA^a^, ω3 PUFA-enriched diet^b^, DHA-enriched diet (omegavie DHA90 TG)	BALB/c and C57BL/6 mice MU spleen and bone marrow DC	↓ T cell activation ↑ Th2 lymphocyte polarization ↓ Th17 lymphocyte polarization ↑ expression and generation of T_reg_	Interference with STAT3 signaling pathway *via* enhancement of PPARγ-induced SOCS3	(43–51)
		↓ T_reg_ suppressive and migratory function ↓ DC activation ↓ DC-mediated T cell activation ↑ DC-mediated T_reg_ expansion	M2 Mφ mediated	

*The table shows a summary of the main publications on the immunomodulatory effects of ω3 PUFA in monocyte/macrophages and T lymphocytes, including both in vitro studies and in vivo animal models*.

*MU, murine; HU, human; Mφ, macrophage; DC, dendritic cell; HFD, high fat diet; GPR120, G protein-coupled receptor 120*.

*^a^Chemically synthesized*.

*^b^ω3 PUFA source: perilla seed oil, menhaden fish oil, and EPAX oil (EPAX-7010) containing ~85% ω3 PUFA (70% EPA, 12% DHA)*.

## Protective Anti-Inflammatory Effects of ω3 PUFA in Colorectal Cancer

Considerable debate still exists on the mechanisms underlying the protective effects exerted by the ω3 PUFA on the establishment/progression of inflammation and cancer (29). Furthermore, the precise mechanism that operates at specific body district, such as the intestine, remains to be defined. Several mechanisms have been proposed to explain the capacity of ω3 PUFA to interfere with important steps of CRC carcinogenesis including alterations of the cellular redox state and modulation of membrane dynamics, and surface receptor function which regulate cell proliferation and apoptosis. Due to the close association of CRC with inflammation, the capacity of ω3 PUFA to modulate some aspects of inflammation and immune response is becoming increasingly important. In this regard, it is of interest that ω3 PUFA inhibit agonist-induced activation of pattern recognition receptors, thus dampening inflammation (52). However, only few studies focused on the role of PUFA-mediated immunomodulatory effects in the therapy of CRC.

Due to the abundance of double bonds, EPA and DHA are susceptible to oxidation and can undergo spontaneous non-enzymatic peroxidation or enzymatic oxidation giving rise to a variety of bioactive lipids that induce inflammation, tumorigenesis, and thrombosis, as well as to mediators with antitumorigenic, pro-resolution properties (53, 54). One of the main mechanisms by which ω3 PUFA exert their anti-inflammatory activity involves enzymatic pathways that are hyper-activated in CRC (55), such as cyclooxygenase (COX) and lipoxygenase (LOX), and leads to prostaglandin E_2_ (PGE_2_) production, a potent pro-inflammatory and procarcinogenic agent. EPA and DHA have been shown to inhibit COX-2 expression in CRC cells (56), and this effect has been attributed to ω3 PUFA capacity to displace ω6 PUFA, in particular AA, from membranes. This metabolic competition is due to structural similarity of EPA and AA, which compete with each other for the same enzymes, COX, and LOX, a very important event in tumor development (57). Furthermore, supplementation with ω3 PUFA significantly decreases AA-derived eicosanoids, while increasing EPA-derived eicosanoids, shifting the balance toward a reduced level of inflammation (58). Interestingly, aspirin, a non-steroidal anti-inflammatory drug, possesses both COX and LOX inhibitory action, and enhances the production of the anti-inflammatory lipid mediator lipoxin A4. Likewise, anti-inflammatory compounds are formed from EPA and DHA by the action of aspirin termed as resolvins (from EPA and DHA) and protectins and maresins from DHA (59, 60). Of note, regular aspirin use substantially reduces the risk of CRC (61). Pro-inflammatory cytokines and tumor-infiltrating myeloid and immune cells play critical roles in almost every developmental stages of inflammation-related cancers, from initiation, promotion, and progression to malignant metastasis (1). A recently published meta-analysis of the ω3 PUFA effects on inflammatory markers highlighted a decreased level of some inflammatory mediators (cytokines or acute phase proteins) in CRC patients who received ω3 PUFA supplementation (26). Likewise, a recent *in vitro* study described a new anti-inflammatory and anticancer properties of DHA. Fluckiger and colleagues reported that inhibition of colon cancer growth and activation of apoptosis by DHA involve autocrine production of TNFα *via* microRNA (miR)-21 (62).

Another mechanism involved in anti-inflammatory and potentially antineoplastic effects of ω3 PUFA regards the possibility that these compounds may modulate epigenetically (through DNA methylation, acetylation modifications, and miR gene regulation) the expression of crucial genes involved in cellular processes associated with colorectal carcinogenesis (63–65). More recently, the epigenetic regulation of gene expression and the polarization of macrophages toward pro-resolving M2 phenotype have emerged as novel mechanisms explaining the antineoplastic effects of ω3 PUFA at the colon level, although additional studies are needed for a deeper comprehension of this issue (30). Finally, the capacity of ω3 PUFA to modulate inflammatory pathways and to generate lipid mediators critical for the resolution of inflammation (e.g., resolvins, protectins, and maresins) has gained attention as a main mechanism involved in the beneficial effects of ω3 PUFA against CRC (66).

## ω3 PUFA as Potential Adjuvants in the Therapy of CRC

Fatty acids may influence carcinogenesis through various mechanisms. EPA and DHA are incorporated into cellular membranes and through the production of lipid mediators and may exert anticancer properties by affecting gene expression or activating signal transduction molecules involved in the control of cell proliferation, differentiation, apoptosis, and metastasis. These properties of ω3 PUFA suggest that they will have important therapeutic potential in cancer management.

Patients undergoing surgery are at risk of developing complications in the postoperative period partly caused by changes in the immune response following surgery (67). Thus, initially, a hyper-inflammatory response followed by a phase of relative immune incompetence occurs in relation to major surgery (68). Patients who undergo surgery for CRC have a 30% risk of infectious complications, and anastomotic leakages are seen in as many as 15% of patients (69).

Initial studies indicated that administration of PUFA-enriched diets leads to increased incorporation of EPA and DHA not only in liver and gut mucosa tissue but also in tumor tissue in patients with solid tumors of the upper gastrointestinal tract (70). This observation suggested that preoperative administration of oral PUFA-enriched diets could have an impact on the postoperative inflammatory response after major abdominal surgery. Subsequently, in a prospective randomized, double-blind, single-center, placebo-controlled study, it was demonstrated a higher total marine ω3 PUFA content in the colonic mucosa, but not in the muscular layer, after 7 days of oral EPA + DHA supplementation to patients admitted for elective CRC surgery (71). Furthermore, providing ω3 PUFA daily for 7 days before surgery resulted in a significant decrease in the formation of the pro-inflammatory leukotriene B4 (LTB4) from neutrophils with a simultaneous increased production of LTB5. However, the clinical consequences of these changes are still unknown as no correlations between values of LTB4 or LTB4/LTB5, and postoperative complication rates were found (72). Likewise, the higher incorporation of EPA, DHA, and docosapentaenoic acid in granulocytes of the ω3 PUFA-enriched supplement group of CRC patients was not associated with improved postoperative outcomes (69). Thus, the effects of ω3 PUFA on preoperative complications need to be investigated in larger trials and for a longer period (months) of ω3 PUFA intake to clearly establish whether LTB formation from activated neutrophils is/is not an important determinant of surgical complications.

Several studies highlighted the beneficial effect of ω3 PUFA as chemopreventive and chemotherapeutic agents in the treatment of several chronic pathologies including cancer providing evidence for a potential use of these compounds to enhance chemo/radiotherapy efficacy and reduce the risk of tumor recurrence (73). Furthermore, several studies demonstrated that EPA and DHA, having immunomodulatory capacity, act to reduce inflammation, even when associated with chemo/radiotherapy immune suppression in lung (74, 75), and in other cancers (73).

In particular, Xue and colleagues evaluated whether ω3 PUFA supplementation could affect the efficacy of a cyclical combination treatment with Irinotecan (CPT-11)/5-fluorouracil (5-FU) in a rat CRC model. Administration of a diet enriched with ω3 PUFA prior to initiating chemotherapy, inhibited tumor growth, while, during chemotherapy, ω3 PUFA-enriched diet enhanced tumor chemosensitivity and reduced body weight loss with respect to control diet fed rats (76). Other studies addressed the issue of whether supplementation with ω3 PUFA to CRC patients undergoing chemotherapy could induce changes in inflammation markers, such as the ratio of C reactive protein (CRP) to albumin, considered a relevant clinical/inflammatory marker. In this respect, some clinical trials demonstrated that EPA and DHA supplementation during chemotherapeutic treatments improved CRP values, CRP/albumin status, and positively modulated the nutritional status of CRC patients (77, 78). Likewise, evaluation of clinical outcomes during and after chemotherapy in CRC patients supplemented with fish oil containing EPA and DHA during the first 9 weeks of treatment unraveled that the time to tumor progression was significantly longer in the supplemented with respect to the control group (79). Interestingly, a recent phase II double-blind randomized, placebo-controlled trial of EPA administration to advanced CRC patients undergoing liver resection surgery demonstrated that EPA is incorporated into secondary CRC tissue and has systemic anti-inflammatory activity. In particular, a clear-cut decrease of the urinary metabolite PGE-M, reflecting systemic PGE_2_ levels, as well as a reversible reduction in NF-kB binding in peripheral blood leukocytes was observed in EPA-treated patients with respect to the placebo group (80). Overall, these observations that supplementation may represent an innovative combination therapy strategy contributing to delay tumor progression and likely enhancing the antineoplastic activity of chemotherapeutic drugs. The design and main hallmarks of clinical trials performed on CRC patients supplemented with ω3 PUFA, described above, are summarized in Table 2.

**Table 2:** **Clinical effects of ω3 PUFA in CRC patients**.

ω3 PUFA daily treatment	Surgery	*n* Subjects	Timing/duration	Chemotherapy	Observed effect	Outcome measures	Reference
1 g DHA + 2 g EPA orally	Yes	148	7 days pre-surgery	No	Potential beneficial effect on local immune function	ω3 PUFA content in the colonic mucosa and muscular layer	(71)
1 g DHA + 2 g EPA orally	Yes	148	7 days pre-surgery	No	Anti-inflammatory effects	Inflammatory markers	(72)
					5-HEPE ↑		
					LTB4/%-HETE ↓		
1 g DHA + 2 g EPA orally	Yes	148	7 days pre- and 7 days post-surgery	No	No significant difference between group in infectious or non-infectious post-operative complications	Levels of ω3 PUFA into granulocytes	(69)
0.6 g EPA/DHA (fish oil) orally	No	23	63 days	5-FU + irinotecan + folinic acid	Positive modulation of nutritional status. CRP/albumin ↓	Evaluation of nutritional status and inflammatory markers	(77)
0.6 g EPA/DHA (fish oil) orally	No	11	63 days	CAPE + OXA + 5-FU + leucovorin	Improved CRP values, CRP/albumin status, plasma fatty acid profile, and potentially prevented weight loss during treatment	Evaluation of inflammatory markers and nutritional status	(78)
0.6 g EPA/DHA (fish oil) orally	No	30	63 days	Standard chemotherapy	Delayed tumor progression time by enhancing the antineoplastic action of chemotherapy	Evaluation of tumor progression time and CEA values after chemotherapy	(79)
2 g EPA-FFA^a^ orally	Yes	88	Median 30 days pre-operative	No	Preoperative treatment may have prolonged benefit on post-operative overall and disease-free survival	Ki67, CD31, and PGE2 levels. Survival statistical analysis (overall and disease-free survival)	(80)

*^a^ALFA (SLA Pharma AG, Liestel, Switzerland)*.

*5-HEPE, 5-hydroxyeicosatetraenoic acid; LTB4, leukotriene B4; 5-HETE, 5-hydroxyeicosatetraenoic acid; 5-FU, 5-fluorouracil; CAPE, capecitabine (Xeloda); OXA, oxaloplatin; CRP, C reactive protein; CEA, carcinoembryonic antigen; PGE2, prostaglandin E.2*.

In the last years, many studies have highlighted the presence of cancer stem cells (CSC) in most tumors, including CRC. These cells are considered responsible for tumor relapse and resistance to conventional therapies (81). Thus, novel anticancer strategies have been designed to selectively target CSC, and to this purpose, natural compounds, including ω3 PUFA, might have a relevant role. In colon cancer stem-like cells (CSLC), De Carlo et al. showed that EPA has a pre-differentiating effect and this finding could, at least in part, explain the increased cellular sensitivity to 5-FU (82). In keeping with these observations, Yang and colleagues demonstrated that EPA and DHA, in a single or combined treatment, enhanced the chemotherapeutic sensitivity effect of 5-FU and mitomycin C by inducing apoptotic cell death of CSLC (83).

## Conclusion

Although the use of ω3 PUFA as potential chemopreventive and chemotherapeutic agents has been frequently proposed in cancer treatment, the evidence achieved points to these compounds as important agents for combined strategies against cancer. In particular, despite some contradictory results, several experimental and clinical data strongly support a role of ω3 PUFA as adjuvants able to counteract the occurrence of inflammatory processes during CRC treatment as well as to increase chemotherapy efficacy or to decrease its toxicity. However, none of the studies has taken into consideration the initial status of the patients prior to PUFA supplementation or dietary advice, a relevant issue to better exploit the preventive/therapeutic/adjuvant potential of these compounds. Further and deepened studies are thus needed to characterize the real ω3 PUFA effectiveness and mechanisms of actions.

## Author Contributions

All authors listed have made substantial, direct, and intellectual contribution to the work and approved it for publication.

## Conflict of Interest Statement

The authors declare that the research was conducted in the absence of any commercial or financial relationships that could be construed as a potential conflict of interest.
